# Evaluation of risk factors for development of severe hyperbilirubinemia in term and near term infants in Turkey

**DOI:** 10.12669/pjms.305.5080

**Published:** 2014

**Authors:** Ali Bulbul, Nihal Cayonu, Merve Emecen Sanli, Sinan Uslu

**Affiliations:** 1 Ali Bulbul, MD, Department of Pediatrics, Division of Neonatology, Sisli Hamidiye Etfal Children Hospital, Istanbul, Turkey.; 2 Nihal Cayonu, MD, Department of Pediatrics, Division of Neonatology, Sisli Hamidiye Etfal Children Hospital, Istanbul, Turkey.; 3 Merve Emecen Sanli, MD, Department of Pediatrics, Division of Neonatology, Sisli Hamidiye Etfal Children Hospital, Istanbul, Turkey.; 4 Sinan Uslu, MD, Department of Pediatrics, Division of Neonatology, Sisli Hamidiye Etfal Children Hospital, Istanbul, Turkey.

**Keywords:** Feeding, Hyperbilirubinemia, Newborn, Risk factors

## Abstract

***Objective:*** To determine clinical features, etiology and risk factors in term and near term newborns with severe hyperbilirubinemia.

***Methods:*** During ten years period (2000 - 2009), infants of ≥ 35 gestational weeks who received phototherapy were evaluated retrospectively. The study population was divided into two groups and clinical features, etiology and risk factors were compared. Group 1 defined by those who had bilirubin level ≥25 mg/dl (severe hyperbilirubinemia) and group 2 defined by bilirubin level <25 mg/dl.

***Results:*** During the study period 1335 babies were evaluated. Severe hyperbilirubinemia was found in 137 (10.3%) patients. Total serum bilirubin level was 29.7±4.7 mg/dl in group 1 and 18.9±3.5 mg/dl in group 2. Pathological weight loss, vaginal delivery and supplementary feeding were identified as significant risk factors for development of severe hyperbilirubinemia (p <0.001, p <0.001 and p = 0.04, respectively). The time at recognition of jaundice by family and postnatal age at admission were significantly higher in group 1. The ratios of previous sibling received phototherapy and being the second child or after were found higher in group 1.

*Conclusion:* Pathological weight loss, vaginal delivery and supplementary feeding were determined as risk factors for development of severe hyperbilirubinemia. The newborns with severe hyperbilirubinemia had late recognition of jaundice and admission to hospital by their families.

## INTRODUCTION

Neonatal jaundice remains the most common and probably often preventable problem in full-term and near-term infants during the early postnatal period. In both developed and developing countries like Turkey the most common cause of infant readmission is hyperbilirubinemia, especially severe hyperbilirubinemia.^1^^-^^4^ Long-term effects of severe hyperbilirubinemia, including kernicterus, were thought to be rare since the advance of exchange transfusion, maternal rhesus immunoglobulin prophylaxis and phototherapy.^4^^-^6 In the world, reported patients of kernicterus are mostly from the United States (27 %), Singapore (19 %) and Turkey (16 %).^7^ Considering the frequency of kernicterus in our country (the third country all over the world), it is clear that further studies concentrating on the etiology and treatment outcomes of neonatal jaundice are necessary.

A total serum bilirubin level of more than 25 mg/dl is accepted as severe hyperbilirubinemia since an infant with this degree of jaundice is thought to be at high risk of kernicterus.^8^ Risk factors recognized to be associated with severe hyperbilirubinemia in newborns have included rhesus and ABO incompatibility, as well as glucose-6-phosphate dehydrogenase (G6PD) deficiency, jaundice in the first 24 hours of life, jaundice noted before discharge from hospital, previous sibling received phototherapy, near - term gestational age of 35–36 weeks, Asian race and the presence of bruising or cephal hematoma.^3^^,^^4^^,^^9^^,^^10^ To provide appropriate epidemiologic data, it is necessary to document the incidence of kernicterus in the newborn population, the incidence of other adverse effects attributable to hyperbilirubinemia and its management, and the number of infants whose TSB (total serum bilirubin) levels exceed 25 or 30 mg/dl.^5^

This study aimed to compare the etiologies and risk factors of hyperbilirubinemia between babies whose TSB levels were ≥ 25 mg/dl and < 25 mg/dl.

## METHODS

***Study design: ***This study was a retrospective medical record review. The records of all newborns admitted to Sisli Hamidiye Etfal Children Hospital, Neonatology Department, from January 2000 to December 2009 were reviewed. The records of newborns with ≥ 35 gestational weeks and who received phototherapy with diagnosis of hyperbilirubinemia were evaluated. The study protocol was approved by the ethical committee of the Sisli Hamidiye Etfal Children Hospital. The study population was divided into two groups according to highest bilirubin level detected in neonatal period (first 28 days of life). The group 1 consisted of newborns whose bilirubin levels were ≥ 25 mg/dl and the group 2 was whose bilirubin levels were < 25 mg/dl. Demographic features, physical examination findings and the risk factors of jaundice were compared between the two groups. Demographic features and all laboratory results were collected from the registration medical records. Gestational age was determined according to the last menstrual period and if it was not known and admission to hospital was in the first 48 hours of life, it was calculated using the Dubowitz scoring system.^11^ The decision to proceed with phototherapy and exchange transfusion was undertaken according to the guidelines proposed by the American Academy of Pediatrics (AAP).^5^^,^^12^

***Etiologic investigations: ***According to our protocol, the following etiologic investigations were performed on all newborns as a baseline: weight loss at admission, mother – baby’s blood group, complete blood count, total and direct serum bilirubin, peripheral blood smear, reticulocyte count, direct Coombs test, thyroid hormone levels, blood culture. The causes of jaundice reported in the records were classified in the following way: “Rh sensitization” was defined as jaundice in Rh-positive newborns with Rh - negative mothers. ABO disease was defined as jaundice in A or B group newborns with group 0 mothers. The etiology of which could not have been clarified by the above mentioned analyses, were further investigated for serum levels of G6PD and pyruvate kinase, tandem mass spectrometry (MS) and “TORCH”; and for urinalysis, culture and reducing substances. Abdominal ultrasonography was performed to eliminate hematoma in cases without hemolysis. Blood and/or urine culture positivity was assessed as proven sepsis or urinary tract infection. 

**Excluding criterias from the study:**

1. Newborns whose gestational age were < 35 weeks
2. Newborns who presented with severe asphyxia, infections, abnormal direct serum bilirubin values and congenital major malformations.
3. Newborns whose records were incomplete

***Type of feeding: ***Breastfeeding refers to infants who were exclusively breastfed with no supplementation of water or formula at any time. Supplementary feeding refers to infants who were breastfed and received additional formula supplements. Formula feeding refers to infants who were exclusively bottle-fed because their mothers presented some pathologic conditions that leads to contraindications to breastfeeding or declined to breastfeed. 

***Pathological weight loss: *** In neonatal period, if infant’s weight loss from birth was **>** 12 % or there is clinical or biochemical evidence of dehydration, it is accepted as pathological weight loss and *calculated as (birth weight - readmission weight / birth weight) x 100.*

***Statistical analyses*** ***:*** SPSS 11.0 software was used for statistical analysis. In the study the demographic features were evaluated by using “descriptive” statistical analysis. Student t test, Chi-square and Fisher’s exact test were used to compare qualitative and quantitative data. The relationship between feeding types and serum bilirubin levels were evaluated by ANOVA test. Statistical signifance was determined as p < 0.05 level.

## RESULTS

During the study period 1570 newborns with gestational age ≥ 35 weeks who were admitted to neonatal intensive care units with jaundice were evaluated. Thirty four newborns had congenital anomalies, and 199 had deficient data in records, so from both groups, in total 235 newborns were excluded and the study was finally completed with 1335 newborns. The demographic features of the patients are presented in Table-I. The relationship of the first admission bilirubin levels and the demographic features of all the patients are shown in Table-II. 

In evaluation of all patients, according to gender; mean TSB levels were found 19.5 ± 4.8 mg/dl in male babies and 18.8 ± 4.6 mg/dl in female babies. Mean TSB level were found 19.6 ± 5.2 mg/dl in normal spontaneous delivery (NSD), 18.2 ± 3.7 cesearean section and 21.2 ± 2.0 mg/dl in vacuum delivery. In male newborns and vacuum deliveries bilirubin level was found to be higher than the others (p: 0.016 and p < 0.001, respectively). 

Severe hyperbilirubinemia (TSB ≥ 25 mg/dl) was revealed in 137 newborns (10.3%). Comparison of the demographic features of the groups is presented in Table-III. The gender had no effect on the occurence of severe hyperbilirubinemia, however the percentage of severe hyperbilirubinemia was significantly higher at vaginal delivery (p < 0.001).

In severe hyperbilirubinemia group there was no difference between the newborns who were on breastfeeding and formula feeding [OR = 1.9, 95% CI= 0.9 – 3.6, p=0.06], however there was a significant difference between the breastfeeding group and the supplementary feeding group [0R = 1.4, 95% CI =1.01 – 2.1, p=0.04]. It was found that severe hyperbilirubinemia was 1.4 times higher in the supplementary feeding group. Jaundice was common in breastfed newborns but it was revealed that breastfeding had no effect on development of severe hyperbilirubinemia.

In all groups, pathological weight loss was found in 107 (8%) newborns. In 81 of them TSB levels were < 25 mg/dl and TSB levels were ≥ 25 mg/dl in 26. Severe hyperbilirubinemia was common and the level of TSB was higher in the pathological weight loss group [OR = 3.6, 95% CI=2.26 – 5.84, p < 0.001]. In the second child, risk of development severe hyperbilirubinemia was found to be higher than the first child (p < 0.002) (Table-III). In the severe hyperbilirubinemia group the ratio of previous sibling who recieved phototherapy, the notification time of jaundice by family and postnatal age at time of admission were found statistically higher than other group (Table-III). In the severe hyperbilirubinemia group jaundice was noticed later than the other group [mean difference - 0.5 day, 95% CI, - 0.1 to - 0.16, p=0.006] and this group was also brought to the hospital later [mean difference - 1.09 day, 95% CI, - 1.7 to - 0.4, p < 0.001].

The etiologic factors of hyperbilirubinemia in both two groups are shown in Table-IV. Pathological weight loss and sepsis were diagnosed more in the severe hyperbilirubinemia group (p < 0.001). No etiologic factor was found in 53.9 % of all infants and there was no difference between the two groups in this respect. 

In all cases 96 (7.8%) newborns underwent exchange transfusion, 61 of them were in the severe hyperbilirubinemia group and 35 were in the other group. Clinical findings of acute encephalopathy were present in 16 patients. Symptoms of 9 patients vanished after exchange transfusion, but the other 7 babies manifested signs of kernicterus during their long term follow-up. All these 7 babies were in severe hyperbilirubinemia group and median TSB level was found as 36.5 mg/dl (min: 31.1 mg/dl, max: 41.4 mg/dl). 

## DISCUSSION

Jaundice is the most common issue in the neonatal period. It is seen in 60 % of term newborns and 5 – 10 % of these newborns with elevated bilirubin levels required admission to hospital and treatment.^7^ In studies to determine which newborns will need treatment for jaundice; early discharge from hospital (both mother and baby), being the first child of the family, male gender, breastfeeding and pathological weight loss have been reported as risk factors.^13^^,^^14^ It is known that indirect hyperbilirubinemia is seen more commonly in the babies of mothers who have no sufficent or suitable support for breastfeeding.^13^ However the common aspect is that jaundice has an ethnic, cultural and geographic distrubution so every country should improve their follow up systems.^6^^,^^15^^,^^16^ In our study we aimed to determine the risk factors of jaundice in our newborns who had severe hyperbilirubinemia in ten years period.

**Table-I:** Baseline demographic characteristics of the all patients

	***Value***	***Range***
Number of patients,	1335	-
Gestational age, weeks	38.6 ± 1.0	35 - 42
Birth weight, g	3082 ± 533	1500 - 5195
Body weight on admission, g	2977 ± 525	1440 - 5110
Admission TSB, mg/dl	19.26 ± 4.80	8 - 46.5
Time jaundice noticed, days	3.5 ± 2.38	1 - 26
Postnatal age at time of admission, days	5.3 ± 3.7	1 - 28
Hospitalization duration, days	4.1 ± 3.0	1 - 34
Previous sibling received phototherapy, n (%)	68 (5.1)	

* Values are given as mean ± standard deviation. TSB: total serum bilirubin.

**Table-II:** The relation between demographic features and first admission bilirubin levels in all patients

	*** n ***	***%***	* ***TSB, mg/dl***	***p***
Number of patients,	1335			
***Gender ***				*0.016*
Male	769	57.6	19.5 ± 4.8	
Female	566	42.6	18.8 ± 4.6	
***Methods of Delivery ***				< 0.001
NSD	837	62.7	19.6 ± 5.2	
Cesarean section	445	33.3	18.2 ± 3.7	
Vacuum	53	4	21.2 ± 2.0	
***Type of feeding ***				0.246
Breastfeeding	805	60.3	19.1 ± 4.3	
Formula	70	5.2	19.9 ± 6.7	
Supplementary	460	34.5	19.5 ± 5.2	

TSB: total serum bilirubin, NSD: Normal spontaneous delivery.

* Given as mean ± standard deviation.

**Table-III:** Comparison of the risk factors of hyperbilirubinemia in groups

***Risk faktors***	***Group 1*** ***(TSB ≥ 25 mg/dl)***	***Group 2*** ***(TSB < 25 mg/dl)***	***p***
Number of patients, n	137	1198	
*Gestational age, weeks	38.5 ± 1.1	38.6 ± 1.0	NS
*Birth weight, g	3146 ± 526	3075 ± 534	NS
*Body weight on admission, g	2928 ± 507	2983 ± 527	NS
*Admission TSB, mg/dl	29.7 ± 4.7	18.9 ± 3.5	< 0.001
***Gender ***			
Male, n (%) Female, n (%)	85 (62) 52 (38)	684 (57.1) 514 (42.9)	NS
***Methods of Delivery, n (%) ***			
NSD Cesarean section Vacuum	111 (81) 21 (15.3) 5 (3.7)	726 (60.6) 424 (35.4) 48 (3.6)	< 0.001
***Type of feeding, n (%) ***			
Breastfeeding Formula Supplementary	70 (51.1) 11 (8) 56 (40.9)	735 (61.4) 58 (14.8 ) 405 (33.8 )	0.04
***Pathological weight loss, n (%) ***			
Present Absent	26 (20.4) 109 (79.6)	81 (6.6) 1119 (93.4)	< 0.001
*Which child of family, n (%)*			
First child Second child or after	53 (38.7) 84 (60.3)	640 (53.4) 558 (46.6)	NS 0.002
Previous sibling received phototherapy, n (%)	29 (21.1)	39 (3.3)	< 0.001
*Time jaundice noticed, days	4.1 ± 2.4	3.5 ± 2.3	0.006
*Postnatal age at time of admission, days	6.2 ± 3.6	5.1 ± 3.7	< 0.001

*NS: Non specific*, NSD: Normal spontaneous delivery.

* *Values given as mean ± standard deviation*

**Table-IV:** Etiology of hyperbilirubinemia in newborns with severe and non-severe hyperbilirubinemia

	***Group 1*** ***TSB ≥ 25 mg/dl*** ***n : 137***		***Group 2*** ***TSB < 25 mg/dl*** ***n : 1198***		***p***
Etiology of hyperbilirubinemia	n	%	n	%	
ABO incompatibility	31	22.6	262	21.9	NS
Rh sensitization	7	5.1	63	5.3	NS
ABO incompatibility + Rh sensitization	4	2.9	24	2	NS
Other blood antigen sensitization	-	-	4	0.3	
G6PD deficiency	1	0.7	7	0.6	NS
Proven sepsis	13	9.5	32	2.7	0.001
Urinary tract infection	-	-	9	0.8	
Hypothyroidism	-	-	7	0.6	
Pathological weight loss	29	21.2	78	6.5	0.001
Previous sibling received phototherapy, n (%)	29	21.1	39	3.3	< 0.001
*Others	1	0.7	35	2.9	NS
No etiologic factor determined	51	37.2	668	55.8	NS

G6PD: Glucose-6-phosphate dehydrogenase*, NS: Non specific*

* Baby of diabetic mothers, Small for gestational age, polyctemia, cephal hematoma etc.

It has been reported that male gender is a risk factor for severe hyperbilirubinemia. Newman et al.^17^ reported that male gender was a risk factor for TSB levels to be ≥ 25 mg/dl although Chou and et al.^18^ reported this TSB level to be ≥ 20 mg/dl. In our study also male gender has a relationship with hyperbilirubinemia, the bilirubin levels were higher in males than females. Although male gender was more common in the severe hyperbilirubinemia group it was not found to be a risk factor for the higher bilirubin levels statistically.

In the first child of the family neonatal jaundice is seen more often because of family inexperience of feeding and caring for the baby and insufficient lactation.^3^^,^^19^ However it has been reported that in healthy term newborns being the first child of the family is not a risk factor for severe hyperbilirubinemia.^3^ In our study jaundice was seen more often in the first child but it was established that being the first child was not a risk factor for the higher bilirubin levels. Contrarily 60 % of the newborns who had severe hyperbilirubinemia were the second child or after. For the severe hyperbilirubinemia the history of previous siblings received phototherapy has been accepted as a risk factor.^10^^,^^16^ Our results were consistent with the literature. In conclusion especially being the second child or after and having a history of previous siblings received phototherapy are the prominent risk factors for development of severe hyperbilirubinemia.

 It has been reported that 58 - 81.4 % of newborns treated with severe hyperbilirubinemia has been only breastfeeding or predominantly brestfeeding.^16^^,^^18^^,^^20^ The percentage of moderate hyperbilirubinemia (TSB > 12 mg/dl) was found to be 14 % in breastfeeding newborns and 4 % in formula feeding; the percentage of severe hyperbilirubinemia (TSB > 15 mg/dl) was 2 % in breastfeeding newborns and 0.3 % in formula feeding newborns.^21^ On the other hand a study of Bertini et al.^15^ has reported that supplementary feeding was an important risk factor for the development of severe hyperbilirubinemia. In our study, we found that the risk of severe hyperbilirubinemia was 1.4 times higher in the supplementary feeding group than breastfeeding group. As a reason of this condition it was thought that the newborns in the supplementary feeding group had insufficient breastmilk so the families preferred to supplement the breastfeeding with formula but still these babies did not have sufficient feeding thus severe hyperbilirubinemia occured with high bilirubin levels.

Pathological weight loss is a risk factor for development of severe hyperbilirubinemia.^3^^,^^5^^,^^15^ Weight loss demonstrates insufficient feeding of the babies and indirectly increases enterohepatic circulation of the bilirubin. Niestjl et al.^22^ emphasized that babies with 5% weight loss should be breastfed more often and that babies with more than 10 % weight loss should be initated supplementary feeding to prevent hyperbilirubinemia. The study of Ebbesen^23^ which compared bilirubin levels between twin babies, declared that there is a direct proportion between high bilirubin levels and weight loss. This study showed that in twins who had the same hereditary and environmental factors weight loss was a risk factor for the high bilirubin levels. Sgro et al.^16^ determined that in 21.1 % babies who come from homes with severe hyperbilirubinemia had weight loss of more than 10 %. In the other study which was conducted in our hospital it was shown that pathological weight loss was a risk factor for severe hyperbilirubinemia.^4^ In this study, when the two groups were compared it was seen that pathological weight loss was an important risk factor for development of severe hyperbilirubinemia.

Appropriate antenatal follow up of mothers with Rh incompabilities and common Rhogram application to them reduced the exchange transfusion requirements. It has been reported that ABO incompatibility has been the most common cause in patients with severe hyperbilirubinemia who received exchange transfusion.^4^^,^^16^ In our study, ABO incompatibility was found to be the most common etiologic factor in newborns with severe hyperbilirubinemia, which is consistent with the literature. In reports 60 – 70 % of patients with severe hyperbilirubinemia had no etiologic factors found.^4^^,^^16^^,^^24^^,^^25^ In our study we could not determine any etiologic factor in 53.9% of all patients. According to us, it was the main cause of concern about jaundice and its complications. At the present day still half of the babies who are admitted to hospital for the treatment because of the hyperbilirubinemia were previously healthy babies without any problems after delivery.

The other interesting part of our study was the information level of our community concerning jaundice. In all groups although the families noticed the jaundice of the newborns on the third or fourth day of life, they stayed at home for two days and brought the newborns to the hospital on the fifth day of life. Sgro et al.^16^ reported that age of the 66 % of the newborns with severe hyperbilirubinemia when the first admission was 111 hours (5 days). We determined the mean age at first admission to be six days in the severe hyperbilirubinemia group. This age was significantly higher than other group. This means that newborns with severe hyperbilirubinemia were brought to hospital later. For this reason the families must be informed about jaundice and its complications before discharge from the hospital and must be told the importance of early admission to hospital as soon as jaundice is noticed. 

In our study population, in a ten year period, pathological weight loss, vaginal delivery, supplementary feeding and being the second child or after were determined as the risk factors for development of severe hyperbilirubinemia. In the severe hyperbilirubinemia group the time between jaundice first being noticed by the families and admission to hospital was longer. At the present day despite all of the developments, it should be remembered that there is no etiologic factor in half of newborns with hyperbilirubinemia which are admitted to hospital.

## Authors Contribution

AB conceived, designed and did statistical analysis & editing of manuscript.

AB, NC, MES & SU did data collection and manuscript writing.

SU did review and final approval of manuscript.

AB takes the responsibility and is accountable for all aspects of the work in ensuring that questions related to the accuracy or integrity of any part of the work are appropriately investigated and resolved.

## Notes

***Declaration of interest:*** The authors report no conflicts of interest. The authors alone are responsible for the content and writing of the paper.

## Notes

***Funding:*** The authors have indicated they have no financial relationships relevant to this article to disclose.
